# Screening of Salt Stress Responsive Genes in *Brachypodium distachyon* (L.) Beauv. by Transcriptome Analysis

**DOI:** 10.3390/plants9111522

**Published:** 2020-11-09

**Authors:** Xiuxiu Guo, Qingjun Wang, Yuan Liu, Xuejie Zhang, Luoyan Zhang, Shoujin Fan

**Affiliations:** Key Lab of Plant Stress Research, College of Life Science, Shandong Normal University, No. 88 Wenhuadong Road, Jinan 250014, China; 2018010081@stu.sdnu.edu.cn (X.G.); 2018020777@stu.sdnu.edu.cn (Q.W.); 2018020782@stu.sdnu.edu.cn (Y.L.); 109131@sdnu.edu.cn (X.Z.)

**Keywords:** *Brachypodium distachyon*, salt stress, physiological responses, transcriptome analysis, wax biosynthesis

## Abstract

As one of the most common abiotic stresses, salt stress seriously impairs crop yield. *Brachypodium distachyon* (L.) Beauv. is a model species for studying wheat and other grasses. In the present investigation, the physiological responses of *B. distachyon* treated with different concentrations of NaCl for 24 h were measured. Therefore, the control and the seedlings of *B. distachyon* treated with 200 mM NaCl for 24 h were selected for transcriptome analysis. Transcriptome differential analysis showed that a total of 4116 differentially expressed genes (DEGs) were recognized, including 3120 upregulated and 996 downregulated ones. GO enrichment assay indicated that some subsets of genes related to the active oxygen scavenging system, osmoregulatory substance metabolism, and abscisic-acid (ABA)-induced stomatal closure were significantly upregulated under salt stress. The MapMan analysis revealed that the upregulated genes were dramatically enriched in wax metabolic pathways. The expressions of transcription factor (TF) family members such as MYB, bHLH, and AP2/ERF were increased under salt stress, regulating the response of plants to salt stress. Collectively, these findings provided valuable insights into the mechanisms underlying the responses of grass crops to salt stress.

## 1. Introduction

Soil salinity has become a worldwide concern in recent years, limiting land application and crop yield. It has been estimated that about 20% of the irrigated farmlands in the world are subjected to salt stress. The halophytes have evolved a variety of molecular, physiological, and biochemical mechanisms to adapt to salt stress, and people have also made efforts to understand the mechanism underlying the salt tolerance of halophytes [1,2,3,4,5,6]. Nevertheless, the majority of crop species are salt-sensitive glycophytes, and improving the salt tolerance of more plants has become an effective scheme to enhance arable area and crop production [7].

Salt stress usually triggers ion/oxidative damage and water deficiency, which has various impacts on plant development and leads to the upregulation of genes associated with salt stress [8]. High salt stress can trigger ion toxicity, reactive oxygen accumulation, and osmotic shock [8]. Under NaCl stress, a large amount of Na^+^ floods into the cell; then, the Na^+^ signal induces the downstream K^+^ transport signal to maintain the relative balance of Na^+^/K^+^ in the cell. The change of Na/K ratio seems to affect the bioenergy process of photosynthesis [9]. The increase of intracellular Ca^2+^ concentration, reactive oxygen species (ROS) production, and cGMP is the early signal in response to salt stress [10,11]. Ca^2+^ signals can activate cation transport channels, such as HAK5 and TPK1, and promote potassium ion influx and sodium ion efflux to maintain cellular ion homeostasis [12,13,14]. Different transcription factors (TFs) such as the MYB family, the bHLH family, and the NAC family play critical roles in transcriptional regulation and signal amplification under salt stress [15,16,17]. An efficient active oxygen scavenging system such as APXs is essential for alleviating oxidative damage to plants [18,19,20]. The accumulation of osmoregulatory substances can maintain the cell pressure and relieve the osmotic shock [21]. Plant hormones have a vital function in the regulation of growth and development. They participate in plants’ responses to various biotic and abiotic stresses such as high salt, drought, and osmotic stresses [22,23,24]. Among them, abscisic acid (ABA) is a critical stress accumulation hormone, and auxin, cytokinin, and brassinosteroids (BRs) are also important anti-stress hormones [25,26,27,28]. Maintaining water equilibrium in plant cells is a vital strategy for plants to respond to salt stress, just as most halophytes have the characteristics of succulents [3]. For non-succulent glycophytes, it is also an important way to alleviate plant ion toxicity and osmotic shock by regulating stomatal opening and wax metabolism of epidermal cells in plant leaves to reduce water loss [29,30].

As a high-quality forage grass and a potential biofuel grass, *Brachypodium distachyon* (L.) Beauv. belongs to the Poaceae family and the Pooideae subfamily, as do *Triticum aestivum* and *Oryza sativa*, and its whole genome has been sequenced [31,32]. It has the advantages of a dwarf phenotype, self-pollination, short life cycle, small genome, easy genetic transformation, and simple growth conditions [33,34]. Compared with rice, *B. distachyon* has a closer relationship with Triticeae crops, and it is a more suitable model system for the genetic analysis of Triticeae crops [35,36]. Phosphorylated proteome analyses have shown that the salt tolerance of *B. distachyon* is enhanced by phosphorylation of related proteins [37]. Moreover, 101 NAC genes have been found in *B. distachyon*, among which BdNAC003 and BdNAC044 are induced by high salt stress [38]. Wang et al. have identified 44 BdSnRKs in *B. distachyon*, and the overexpression of BdSnRK2.9 in tobacco increased the tolerance of tobacco to drought and salt stresses [39]. However, only a few studies related to the salt stress of *B. distachyon* have been reported, and the analysis of the whole transcriptome may provide valuable insights into the mechanism underlying the salt stress response of *B. distachyon*.

Transcriptome sequencing is a common and effective strategy to identify gene expression across the genome of a cell in a specific state, which is conducive to understanding the response mechanism and identifying candidate genes. Transcriptome technology has been used to analyze the salt tolerance of many species, including Dongxiang wild rice [40], wheat [41], and sorghum [42]. The combination analysis of the spatiotemporal expression patterns of genes and corresponding traits has advantages in identifying differentially expressed genes (DEGs) related to salt stress [2,3]. Transcriptome analysis has revealed that polyunsaturated fatty acid metabolism, photosynthesis, and jasmonic acid signal transduction pathways are responsive to wheat salt stress [41]. However, there is no report on the salt tolerant transcriptome of *B. distachyon* yet.

In our current work, we performed Illumina RNA sequencing on the leaves of *B. distachyon* under salt stress. We found that, in addition to the active oxygen scavenging system, production of osmoregulatory substances and regulation of plant hormone signals could alleviate the damage induced by salt stress. Moreover, the regulation of TFs, ABA-signaling-induced stomatal closure, and cuticular waxiness to prevent nonstomatal water loss were important strategies to improve the salt stress tolerance of *B. distachyon*.

## 2. Results

### 2.1. Measurement of Biochemical Parameters

**Figure 1:**
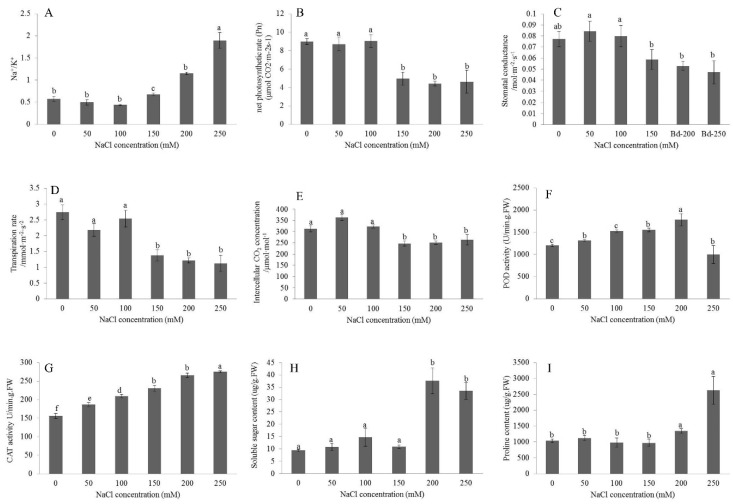
Biochemical parameters of leaf tissue in control group and treated group (200 mM NaCl for 24 h). (**A**–**I**) represented the change of Na^+^/K^+^, net photosynthetic rate (Pn), stomatal conductance (Gs), transpiration rate (Tr), intercellular CO_2_ concentration (Ci), peroxidase (POD) activity, catalase (CAT) activity, soluble sugar content and proline content with NaCl concentration respectively. Most of these data were obtained from the average of three biological replicates, among which the data of photosynthesis indicators Pn, Gs, Tr and Ci were got from the means of six biological replicates. The individual black bars are the means ± SD of three or six measurements. The letters on the black bars represent the significance of the difference.

The physiological responses of the leaves of *B. distachyon* under different NaCl concentration gradients for 24 h were measured. The results showed that the Na^+^/K^+^ ratio gradually increased with the increase of NaCl concentration. When the NaCl concentration reached 200 mM, the Na^+^/K^+^ ratio increased significantly. The photosynthetic performance indicators including Pn, Gs, Tr, and Ci all decreased with the increase of NaCl concentration. When an NaCl concentration of 150 mM was achieved, these indicators tended to stabilize. The activity of POD augmented with the increase of NaCl concentration. When the concentration of NaCl attained 200 mM, the POD activity met the maximum value of 1783.33 U/min·g·FW, and then decreased significantly. The activity of CAT rose with the increase of NaCl concentration and tended to be stable when the concentration of NaCl reached 200 mM. In this study, the changes of two osmotic adjustment substances under salt stress were determined. Under the challenge of low NaCl concentration, the contents of these two substances did not change significantly. The soluble sugar content was significantly increased to 37.6 μg/g·FW under 200 mM NaCl stress. When the NaCl concentration was 250 mM, the soluble sugar content decreased slightly to 33.5 μg/g·FW. The proline content increased slightly under 200 mM NaCl stress. When the NaCl concentration was increased to 250 mM, the proline content increased significantly to 2626.55 μg/g·FW (Figure 1). 

### 2.2. Transcriptome Profiling of B. distachyon

Based on the results of physiological indicators, the leaves under 200 mM NaCl stress for 24 h were selected for transcriptome analysis. A total of 59,206,904, 63,617,088, 57,536,132, 55,294,072, 65,989,512, and 47,315,154 pair-end reads were acquired from three control and three NaCl-exposed samples of *B. distachyon* (Table 1), respectively. Reads were mapped to the reference genome, with a mapping mean of 95.92%, ranging from 95.48 to 96.76% for the six samples (Table 1). Quality score or Q-score is an integer mapping of the probability of base calling errors. The higher the base Q-score is, the more reliable the base recognition is, while the less the possibility of base error detection is. The Q30 values ranged from 94.49% to 95.26%, with an average of 94.97% (Table 1).

### 2.3. DEGs in B. distachyon

**Figure 2:**
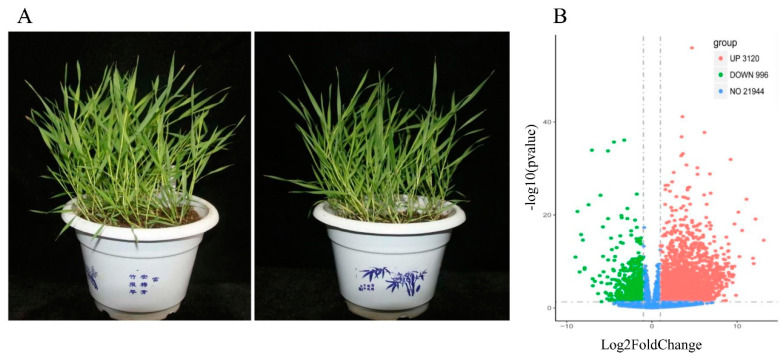
(**A**) 3-week-old seedlings of *B. distachyon*; the left picture was the control and the right picture was the treatment with 200 mM NaCl for 24 h. (**B**) Expression patterns of DEGs identified between 200 mM NaCl treatment group and control group. SALT_S indicated that cells were exposed to 200 mM NaCl for 24 h; CK_S indicated that cells were cultured under the control condition. Red and green dots represent DEGs, and blue dots indicate genes that were not differentially expressed.

The relative gene expression in *B. distachyon* was evaluated by fragments per kilobase of exon model per million reads mapped (FPKM) values under the control or salt stress conditions. The FPKM values for genes identified in the six samples ranged from 0 to 12,225.71, with an average of 27.88. With a screening threshold of |log2 (fold change)| > 1 and *p*-value < 0.05, 4116 genes were selected by comparing the profiles of control and NaCl-exposed samples, including 3120 upregulated and 996 downregulated ones (Figure 2B and Table S1 in Supplementary Materials). Table 2 lists the top 30 upregulated and downregulated genes in *B. distachyon* upon the exposure to 200 mM NaCl.

### 2.4. Gene Ontology (GO) Enrichment Results of DEGs in B. distachyon

The GO database was employed to illustrate the molecular basis of *B. distachyon* leaves under salt stress by characterization of DEGs. We concluded that 182 biological process (BP) terms, such as “plant-type secondary cell wall biogenesis” (GO: 0009834), “hydrogen peroxide catabolic process” (GO: 0042744), and “oxidation-reduction process” (GO: 0055114), were enriched by 3120 upregulated genes with a cutoff of *p* < 0.05 (Table 3 and Table S2). Moreover, 996 downregulated genes were recruited in 121 BP terms, such as “cellular response to heat” (GO: 0034605), “hydrogen peroxide catabolic process” (GO: 0042744), and “plant-type cell wall assembly” (GO: 0071668) (Table 3 and Table S2). Table 3 lists the top 30 BPs enriched by the upregulated and downregulated genes.

**Figure 3:**
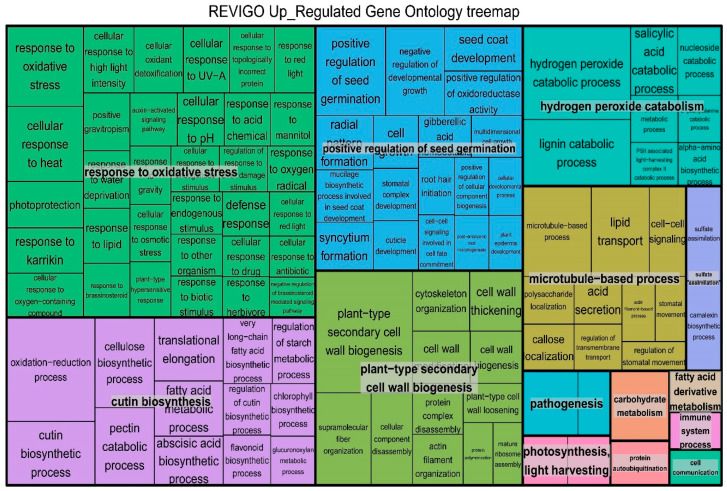
REVIGO analysis results for genes upregulated in *B. distachyon* under salt stress. Each rectangle is a single cluster representative. The representatives are joined into “superclusters” of loosely related terms and visualized with different colors. The sizes of the rectangles were adjusted to reflect the *p*-value of the GO terms calculated by TopGO. In this study, 182 upregulated processes were integrated into 14 groups.

The GO enrichment results were visualized using REVIGO, and the representative subgroups of the terms were identified accordingly. The 182 BPs enriched by upregulated genes were integrated into 14 subgroups (Figure 3), 30 terms were classified into the “response to oxidative stress” group, 18 terms were summarized into the “microtubule-based process” subset, and 16 terms were integrated into the “positive regulation of seed germination” group. In addition, 121 BPs enriched by downregulated genes were integrated into ten subsets (Figure S1), 24 terms were classified into the “cellular response to heat” group, 20 terms were summarized into the “translational elongation” subset, and 12 terms were integrated into the “zinc II ion transmembrane transport” group.

### 2.5. Kyoto Encyclopedia of Genes and Genomes (KEGG) and MapMan Enrichment Results of DEGs in B. distachyon

**Figure 4:**
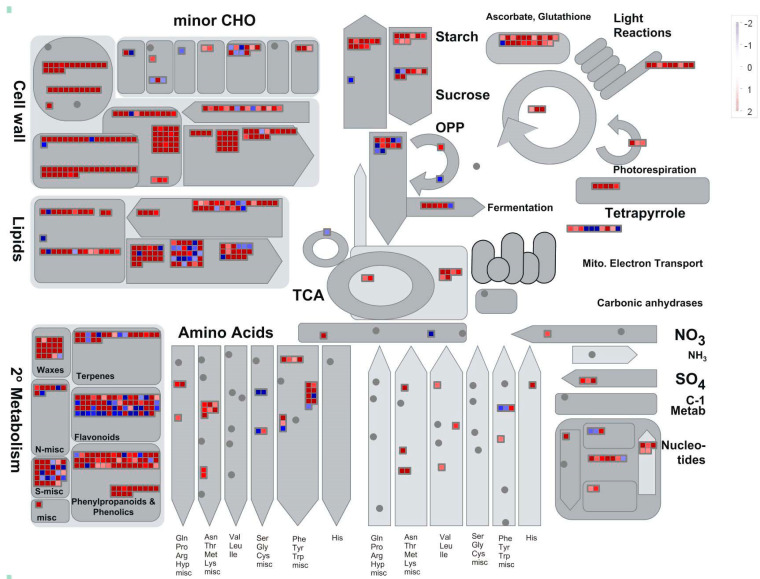
Global view of DEGs involved in diverse metabolic pathways. DEGs were selected for the metabolic pathway analysis using the MapMan software (V3.6.0RC2). The colored boxes indicate the log2 of expression ratio of DEGs. The DEGs could be mapped to 713 pathways by MapMan, of which 88 pathways were filtered and enriched by the dysregulated genes with a cutoff of *p* < 0.05.

In order to more specifically compare the metabolic and regulatory pathways, MapMan was used in the present study. The DEGs were mapped to 713 pathways by MapMan, of which 88 pathways were filtered and enriched by the dysregulated genes with a cutoff of *p* < 0.05 (Figure 4). The genes implicated in “secondary metabolism. Wax”, “cell wall. cell wall proteins”, “starch”, “sucrose” and “redox.ascorbate and glutathione” were overexpressed in *B. distachyon*, while those genes involved in “minor CHO metabolism.sugar alcohols”, “gluconeogenesis/glyoxylate cycl” and “stress.abiotic.heat” were downregulated in *B. distachyon* during salt stress response (Figure 4 and Table S3).

In order to better uncover the responses of plant hormones under salt stress in *B. distachyon*, KEGG enrichment analysis was performed on DEGs. We found that 39 upregulated genes which participated in “plant hormone signal transduction” (ko04075) KEGG pathways were mapped (Table S4), such as the auxin synthesis genes YUCCA family member (YUC11 BRADI2G10302, Log2fc = 4.4035), the key enzyme in the biosynthesis of ABA 9-cis-epoxycarotenoid dioxygenase NCED9 (BRADI1G13760, Log2fc = 1.1422), and brassinosteroid insensitive1 BKI1 (BRADI4G32220, Log2fc = 1.7916) (Figure S2).

### 2.6. The Differentially Expressed TFs

A total of 289 TFs of 30 TF families were differentially expressed in the leaves of *B. distachyon* under salt stress, including 212 upregulated TFs and 77 downregulated TFs (Table S5). Among the upregulated TFs, most upregulated genes belonged to the MYB family, with 39 genes. However, most downregulated TFs belonged to the bHLH family, with 14 genes (Table 4).

### 2.7. Quantitative Real-Time PCR (qRT-PCR) Validation

**Figure 5:**
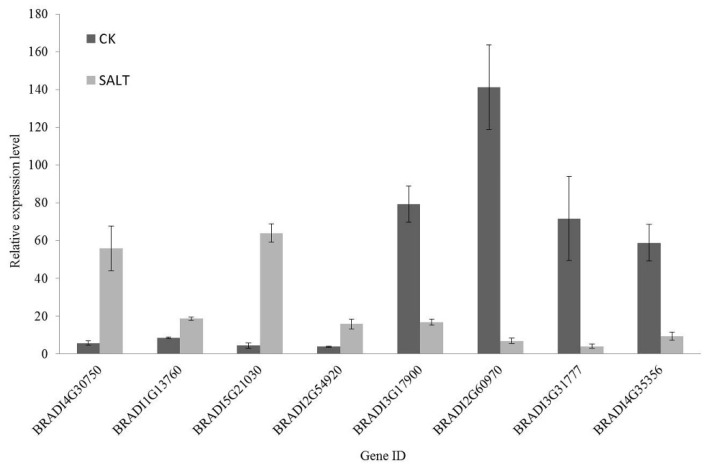
Validation of RNA-Seq results by qRT-PCR using eight *B. distachyon* dysregulated genes, which were randomly selected from all (4116) DEGs. The expression levels of eight selected DEGs under control and salt stress conditions are shown. The grey bars represent the qPCR results of samples under salt stress condition, while the corresponding black bars represent the results of control samples. The individual black bars, representing the qPCR data, are the means ± SD of nine measurements (three technical replicates each for three biological samples).

For the dysregulated genes, qRT-PCR was a frequently used method to validate the RNA-Seq results. Primers were designed spanning exon–exon junctions (Table S6). Although they did not exactly match each other, the expression tendencies of all 8 genes from qRT-PCR were mostly consistent with the Illumina–Solexa RNA sequencing analyses, demonstrating that the RNA-Seq results were reliable (Figure 5). For instance, the ortholog of delta 1-pyrroline-5-carboxylate synthetase B (BRADI2G54920), which was identified as an upregulated unigene by RNA-Seq in the salt-exposed samples (L2fc = 3.8801), was also significantly upregulated according to the qRT-PCR approach (Figure 5).

## 3. Discussion

As an increasingly serious global concern, soil salinization has hindered the growth and development of plants, leading to reduced crop yield production [7]. Although a great deal of evidence has shown the mechanism of salt tolerance in plants, the genetic underpinnings of salt-responding characteristics still remain largely unexplored because of the complexity of the response to abiotic stress [2]. It is an effective strategy to investigate the mechanism underlying the salt tolerance of model plants [7]. *B. distachyon* has the characteristics of model organism, and its whole genome sequencing and annotation have been completed. It is a new model plant for wheat, barley, and several potential latent biofuel grasses [31,34,37]. In this work, the physiological responses of *B. distachyon* under different NaCl concentration stress were determined. The measurement results of sodium-to-potassium ratio, photosynthesis index, POD, CAT, soluble sugar, and soluble protein showed that *B. distachyon* was the most active physiological state under 200 mM NaCl threat. RNA-Seq was employed to explore the response of *B. distachyon* to salt stress. Under NaCl treatment of 200 mM, 4116 DEGs were identified, including 3120 upregulated and 996 downregulated ones. Our results suggested that ABA-signaling-induced stomatal closure and cuticular waxiness to prevent nonstomatal water loss played a critical role in the salt stress process.

Abiotic stress can rapidly increase the Ca^2+^ concentration in cytoplasm. Therefore, Ca^2+^ is considered as the second messenger of the main stress signal [8,43]. The increase of intracellular Ca^2+^ concentration, ROS production and cGMP is the early signal in response to salt stress [10,11]. Annexin AtANN4 mediates the increase in Ca^2+^ induced by salt stress, and it is subsequently phosphorylated by SOS2, attenuating calcium waves, and producing salt-specific calcium signals [44]. The homologous gene of ANN4 (BRADI2G26760, Log2fc = 4.6317) in *B. distachyon* was also upregulated under salt stress. On the one hand, the calcium signals in the cytoplasm cause the ion channels located in the cell membrane to open, resulting in the influx of potassium ions and the outflow of sodium ions [14]. On the other hand, the local vacuole two-pore K^+^ channel 1 (TPK1) induces the release of Ca^2+^ in the vacuole, thereby enhancing the Ca^2+^ signaling [12]. The tpk1 mutant shows higher salt sensitivity [13]. The homologous gene of TPK1 (BRADI2G12740, Log2fc = 3.1521) in *B. distachyon* was also upregulated under salt stress. Cation sodium transporter (HKT), potassium transporter (HAK), and cyclic nucleotide gated ion channel (CNGC) are generally the carriers for Na^+^ and K^+^ [7,45,46,47]. Our findings suggested that the genes encoding HKT, KT, and CNGC were upregulated to uptake more Na^+^ to cytoplasm, including sodium transporter (HKT1, BRADI5G21030, Log2fc = 2.6659), potassium transporter (HAK5, BRADI2G59757, Log2fc = 2.0307), cyclic nucleotide gated ion channel (CNGC4, BRADI2G51836, Log2fc = 3.7076; GLR2.7, BRADI1G46947, Log2fc = 1.9223), and vacuolar ATP synthase subunit A (VHA-A, BRADI3G05590, LogFC = 2.0765). This is consistent with the trend of physiological indicators. Compared with the control group, the Na^+^/K^+^ of *B. distachyon* under 200 mM NaCl stress increased 3.29 times, from 0.577 to 1.895. This is consistent with the research results of Sade et al. in *Brachypodium sylvaticum* [48]. In addition, a number of studies have shown that Cl^-^ carriers located in plasma membrane are upregulated to preserve the charge equilibrium in the cytoplasm during salt stress [5,49,50]. However, in our research, no Cl^-^ carriers were upregulated. 

As oxygen-containing reactive chemical species, ROS play important functions in cellular signaling. The generation of ROS is an early signal for plants to respond to Na^+^ [7,51]. Under salt stress, plants will produce high levels of ROS, which will destroy the redox homeostasis and cause oxidative damage to plant cells [52]. Therefore, it is highly necessary for plants to have an effective active oxygen scavenging system in response to salt stress [20]. Compared with the control group, the contents of POD and CAT in *B. distachyon* under 200 mM NaCl stress both increased by an approximate factor of 1.5. Wang et al. found that under 300 mM NaCl stress, the CAT and POD contents of *Zoysia japonica* both increased by an approximate factor of 3 [1]. Wu et al. also got a similar conclusion in the study of *Fagopyrum tataricum* under salt stress [53]. According to the results of transcriptome sequencing, the GO terms, such as “hydrogen peroxide catabolic process” (GO: 0042744), “positive regulation of oxidoreductase activity” (GO: 0051353), and “superoxide metabolic process” (GO: 0006801), were enriched by upregulated genes. Furthermore, studies on soybean [54], *Salicornia brachiata* [55], *Atriplex centralasiatica* [51], etc. showed that the expressions of key genes related to active oxygen scavenging significantly increased under salt stress. In our conclusion, the representative genes, such as cytosolic ascorbate peroxidase 1 APX1 (BRADI1G65820, Log2fc = 1.6153), ascorbate peroxidase 3 APX3 (BRADI5G03640, Log2fc = 5.2924), and GSTF8 (BRADI1G76080, Log2fc = 9.0475), which are associated with ROS scavenging, were also upregulated. The data obtained from transcriptome sequencing showed that these redox processes and reducing enzymes played a key role in salt tolerance of *B. distachyon* (Tables S1 and S2).

TFs play a fundamental function in plant growth and regulation. Under salt stress, they interact with related proteins to activate or inhibit the transcription of downstream events. Numerous investigations have proved that TFs participate in salt stress response of plants, such as VvbHLH1, OsMYB2, AP2/ERF, bZIP and NAC [15,16,17,56]. The MYB TF family, named after its conserved myb domain, is one of the largest TF families in plants, and the members of this family play an important role in response to hormone stimulation and external environmental stress. In the present study, 36 MYB family TFs were upregulated, while only four were downregulated in NaCl-exposed *B. distachyon*, including the homologous gene of OsMYB91 (BRADI4G03970, Log2fc = 3.0664), which can improve the salt tolerance of rice [57], and the homologous gene of TaMYB30 (BRADI3G37047, Log2fc = 1.8087), which can enhance the salt tolerance of wheat [58]. bHLH is a type of important TF with basic/helix loop helix structure. In our current study, 34 and 14 bHLH family TFs were upregulated and downregulated in NaCl-exposed leaves, respectively, including phytochrome interacting factor 3 (BRADI2G36740, Log2fc = 3.7496) and CIB1 Like protein 2 (BRADI4G19577, Log2fc = 5.6899). The AP2/ERF is a family consisting of plant-specific TFs, including four subfamilies of AP2, RAV, ERF, and dehydration response element binding protein (DREB) [59]. Moreover, 19 differentially expressed TFs, including ethylene response factor 43 (BRADI3G33355, Log2fc = 3.8957) and WIN1/SH N1 (BRADI3G07450, Log2fc = 5.2271), were upregulated. These differentially expressed TFs indicated that TFs played an important part in response of *B. distachyon* to salt stress, which could be helpful in understanding the mechanism of salt tolerance. 

Osmotic stress is one of the important reasons that salt stress causes damage to plants. Plants complete osmotic adjustment to reduce salt damage by selectively absorbing inorganic ions and accumulating organic solutes that are nontoxic to cells. Consequently, osmotic adjustment is an important physiological mechanism for plants to resist salt stress [60]. Soluble sugar and proline are commonly used osmotic adjustment substances in plants. Under 200 mM NaCl stress, the soluble sugar and proline content of *B. distachyon* increased significantly. The content of soluble sugar was 4 times higher than that of the control, while the proline increased from 1047.5969 μg/g·FW to 1346.3178 μg/g·FW. This is consistent with the trend in barley research [61]. The GO enrichment assay indicated that the upregulated genes were recruited to GO: 0010555 (“response to mannitol”), GO: 2000904 (“regulation of starch metabolic process”), GO: 1901607 (“alpha-amino acid biosynthetic process”), and other GO terms. Genes encoding amino acid synthetase, such as Delta 1-pyrroline-5-carboxylate synthetase (P5CS2, BRADI2G54920, Log2fc = 3.8801), which participated in proline biosynthesis and was famous for its osmotic protective function, were upregulated under salt stress; similar findings have been observed under osmotic stress in rice [62]. Raffinose (sucrasylgalactoside oligosaccharide) is a type of water-soluble sugar, which accumulates in plants under abiotic stress. The overexpression of TsGOLS2 increases the tolerance of *Arabidopsis* to high salt and osmotic stresses [63]. Osmotic stress induces the accumulation of raffinose in seedlings of winter vetch, seedlings of pea (L.), and the leaves of sugar beet [21,64,65]. Our transcriptome data indicated that gossypol synthesis gene 1 (RS1, BRADI3G39220, Log2fc = 2.7874) was upregulated (Table S1). These results indicated that these substances might be essential for maintaining the homeostasis of *B. distachyon* under NaCl stress conditions.

**Figure 6:**
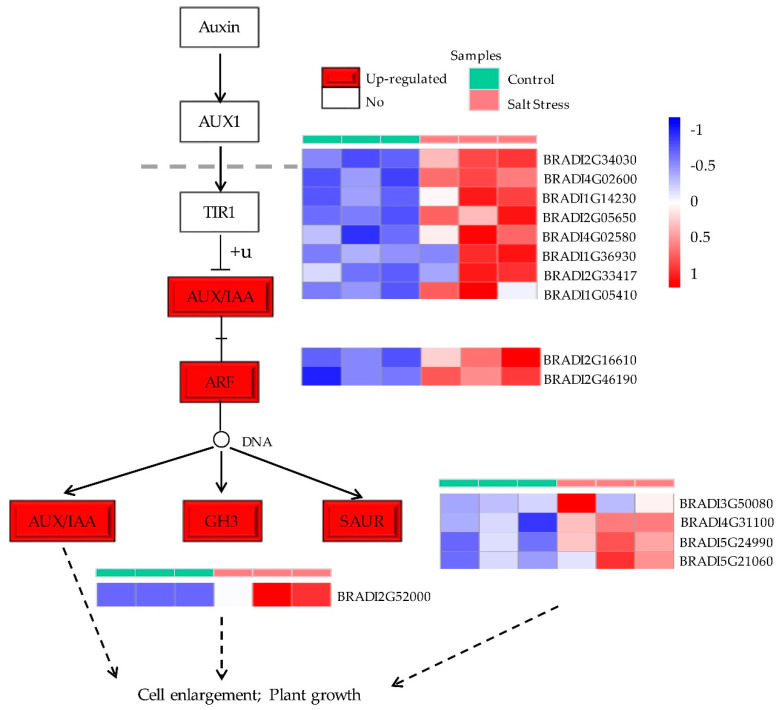
The auxin signal transduction pathway obtained by Kyoto Encyclopedia of Genes and Genomes (KEGG) enrichment analysis. There were 17 DEGs mapped to this pathway. The red boxes indicate that the upregulated genes were enriched in this node. The blank boxes represent nodes with no DEG enrichment.

Plant hormones, especially auxin, ABA, and BRs, have a vital function in regulating plant response to abiotic stress and antioxidants [26,45,66]. Christian et al. compared two varieties of maize with different salt tolerance under salt stress and found that the auxin content increased under salt stress [67]. In our current work, the auxin synthesis gene YUCCA family member (YUC11 BRADI2G10302, Log2fc = 4.4035), aldehyde dehydrogenase (ALDH3F1, BRADI3G50180, Log2fc = 5.0966; ALDH4, BRADI4G41190, Log2fc = 3.7896), AXR3/IAA17 (BRADI2G34030, Log2fc = 1.3116), and SAUR53 (BRADI5G24990, Log2fc = 3.5415) were upregulated under salt stress (Figure 6) [68,69]. Studies on *Dunaliella salina* and *Diaphoreolis viridis* showed that salt stress increased the ABA levels in these two species by 2–3 times [70]. In this study, 9-cis-epoxycarotenoid dioxygenase NCED9 (BRADI1G13760, Log2fc = 1.1422), a fundamental enzyme in the biosynthesis of ABA, was upregulated. Based on the transcriptome data of *B. distachyon* under salt stress, our findings were consistent with the previous data which indicated that the genes related to BRs signal transduction, such as BKI1 (BRADI4G32220, Log2fc = 1.7916), BSK2 (BRADI3G33950, Log2fc = 3.3028), and BZR1 (BRADI1G23550, Log2fc = 3.8252), are upregulated (Figure S2) [71,72,73].

Stomatal opening and wax biosynthesis of epidermal cells in plant leaves alleviate ion-toxicity-induced damage by reducing water loss under salt stress [74,75,76]. One of the physiological responses regulated by ABA is related to stomatal closure, which can prevent excessive transpiration and reduce water loss [25]. The plant-specific actin binding protein SCAB1 (BRADI1G61680, Log2fc = 1.0176) is a positive modulator of ABA-regulated stomatal movements [77]. GHR1 (BRADI1G58260, Log2fc = 8.205514828) participates in the signal transduction of stomatal movement mediated by ABA and H2O2 [78]. The R2R3-type TF MYB60 (BRADI4G43937, Log2fc = 4.8253; BRADI4G22637, Log2fc = 3.2800) induced by early drought stress promotes root growth and increases water absorption by regulating root growth capacity. Otherwise, it has been found that the key gene of waxy CsWAX2 synthesis in cucumber is induced by drought, salt, and ABA stresses [30]. According to our transcriptome data, the GO terms GO: 0010143 (“cutin biosynthetic process”), GO: 1901957 (“regulation of cutin biosynthetic process”), and GO: 0042335 (“cuticle development”) were well collected by upregulated genes. The MapMan displayed that there were 20 genes enriched in the waxy metabolism pathway, of which 19 genes were upregulated and only one gene was downregulated (Table S3). Furthermore, the key enzymes of wax synthesis, such as TF SHN1 (BRADI3G07450, Log2fc = 5.2271), fatty acid hydroxylase CER1 (BRADI3G55100, Log2fc = 5.9220) and long-chain acyl-CoA synthetase 2 (LACS2, BRADI4G16280, Log2fc = 3.4921), were significantly upregulated under salt stress (Table S1). In addition, the energy spectrum analysis of the waxy decoration and waxy particles on the surface of the leaves of *Puccinellia tenuiflora* found that some elements in the wax are the same as those reported in the secretion of the salt glands, indicating that the excessive salt can be discharged from the leaves of *P. tenuiflora* under salt stress [79,80].

## 4. Materials and Methods 

### 4.1. Material Preparation and Salt Stress Treatment

The *B. distachyon* variety Bd21 was used in the present study. Seeds of Bd21 were gifted by Dr. Wu Hongyu from the College of Life Sciences, Shandong Agricultural University. The plump seeds were firstly selected, sterilized using 75% ethanol for 1 min, and then rinsed by sterile water for 3–4 times. The seeds of *B. distachyon* were first germinated in a 9-cm dish with two layers of wet filter paper at room temperature for about 2 days. After 2 weeks of seed vernalization at 4 °C, every 10 plants were transplanted into a plastic basin (14 and 13 cm in diameter and height, respectively), which was filled with matrix and vermiculite (2:1 *v*/*v*). It was grown in a greenhouse under the conditions at a temperature of 20–25 °C, a 16/8-h light/dark cycle, and a relative humidity of about 70%, with the light intensity of 200 μmol photons m^−2^ s^−1^. The plants were daily irrigated using 1/2 Hoagland’s solution.

After 3 weeks, the control group was continuously treated with salt-free 1/2 Hoagland solution according to a previously described method with minor modifications [81]. The treatment groups were treated with 50, 100, 150, 200, and 250 mM NaCl. After 24 h of salt challenge. There were three biological repeats in each group. In order to ensure the accuracy of photosynthetic index data, salt stress treatment and sample harvest were conducted at 10:00 a.m. The samples we harvested were the first and second leaves that were fully expanded above. Two leaves were taken from each sample, and each treatment was performed in three replicates. The control and treated leaves were collected, frozen in liquid nitrogen right away, and then preserved at −80 °C prior to further analysis.

### 4.2. Measurement of Biochemical Parameters

The leaves of the control group and the treatment groups treated with 50, 100, 150, 200, and 250 mM NaCl for 24 h were taken for measurement of physiological parameters. The sodium and potassium contents in the leaves were determined using a flame spectrophotometer (M410, Sherwood, UK) [82]. Photosynthetic parameters including net photosynthetic rate (Pn), stomatal conductance (Gs), transpiration rate (Tr), and intercellular CO_2_ concentration (Ci) were measured using a LI-COR 6400 XT instrument (LI-COR Inc, Lincoln, NE, USA) [83]. The peroxidase (POD) activity of leaves was determined through the guaiacol method [84]. The catalase (CAT) activity of the leaves was analyzed using the ultraviolet absorption method [84,85]. The contents of soluble sugar and proline in leaves were determined through anthrone colorimetry and sulfosalicylic acid colorimetry means, respectively [86,87,88]. Microsoft Excel 2010 software was used to make charts, and SPSS software version 18.0 (SPSS, Inc., Chicago, IL, USA) analysis software was used for the correlation analysis and significant difference test.

### 4.3. Transcriptional Profiling

The leaves of the control group and the treatment group treated with 200 mM NaCl for 24 h were collected. Total RNA was extracted using TRIzol Reagent (Invitrogen, Carlsbad, CA, USA) according to the manufacturer’s procedures. RNA content and integrity were assessed with the RNA Nano 6000 Assay Kit of the Agilent Bioanalyzer 2100 system (Agilent Technologies, Santa Clara, CA, USA) and a NanoDrop 2000 spectrophotometer (Thermo Scientific, Wilmington, NC, USA). Subsequently, transcriptome sequencing was performed. Each experiment was conducted in triplicate. 

For each sample, 1.5 µg RNA was used as input material. The NEBNext^®^ Ultra™ RNA Library Prep Kit for Illumina^®^ (NEB, Ipswich, MA, USA) was adopted to construct the RNA sequencing libraries and index codes were added to attribute sequences to each sample. Briefly, mRNA was further isolated from total RNA using poly-T oligo-attached magnetic beads. To select 150~200-bp cDNA fragments, the AMPure XP system (Beckman Coulter, Beverly, MA, USA) was employed to purify the library fragments. After enrichment and screening by PCR, the products were sequenced using the Illumina HiSeq X platform (Illumina, San Diego, CA, USA). cDNA library generation and PE150 sequencing were performed by Novogene Co., Ltd. The adapter-containing reads were removed, poly-N reads were deleted, and low-quality reads were discarded. Finally, only clean reads were retained. 

The reference genome file and the gene model annotation file of *B. distachyon* were downloaded from ftp://ftp.ensemblgenomes.org/pub/release-39/plants/fasta/brachypodium_distachyon/dna/ and ftp://ftp.ensemblgenomes.org/pub/release-39/plants/gtf/brachypodium_distachyon/Brachypodium_distachyon.v1.0.39.gtf.gz, respectively. Hisat2 v2.0.5 [89] was used to build the index of the reference genome, and the paired-end clean reads were aligned to the reference genome. StringTie13.3b was used to predict the functions of new transcripts [90]. All sequencing data generated in this study were submitted to the NCBI Sequence Read Archive (SRA) database (https://www.ncbi.nlm.nih.gov/sra), with accession No. of PRJNA636626.

### 4.4. Measurement of Gene Expression and Differential Expression Analysis in B. distachyon

The read number mapped to each gene was counted using the feature Counts v1.5.0-p3 [91]. The fragments per kilobase of transcript sequence per millions base pairs sequenced (FPKM) method was selected to calculate the gene expression [92]. The DESeq2 package of R (version: 1.12.0) was used to perform the differential expression analysis of two groups (three biological replicates per condition) [93]. The edgeR package of R V3.18.1 was adopted to conduct the differential expression analysis of two conditions [94]. The screening conditions were |log2 (fold change)| > 1 and *p*-value < 0.05.

### 4.5. Gene Ontology (GO), Kyoto Encyclopedia of Genes and Genomes (KEGG) Enrichment and Differentially Expressed TFs Analysis

The GO annotation file of *B. distachyon* was downloaded from https://bioinformatics.psb.ugent.be/plaza/versions/plaza_v4_monocots/download/index [95]. The topGO package of R was used for the GO enrichment analysis of DEGs [96]. The tool REVIGO was used to identify GO terms into corresponding subgroups and visualize the results [97]. The statistical enrichment of DEGs in KEGG pathways was tested using KOBAS software [98]. The metabolic pathway analysis of the DEGs was carried out by the MapMan software V3.6.0RC2 [99]. The iTAK database (http://itak.feilab.net/cgi-bin/itak/index.cgi) was used for TF analysis [100].

### 4.6. Quantitative Real-Time PCR (qRT-PCR)

qRT-PCR was necessary to validate the expression patterns revealed by the RNA-Seq analysis. AT5G60390, encoding elongation factor-1α (EF-1α), is the most commonly used housekeeping gene for qRT-PCR in plants, and its homolog in *B. distachyon* (BRADI1G06870) was used in this study [101]. Four upregulated genes (BRADI4G30750, BRADI1G13760, BRADI5G21030, and BRADI2G54920) and four downregulated genes (BRADI3G17900, BRADI2G60970, BRADI3G31777, and BRADI4G35356) were randomly chosen for qRT-PCR. The primer5.0 was used to design gene-specific primers used for qRT-PCR (Table S4).

For each sample, 1 μg of RNA was treated with DNaseI, and then cDNA synthesis was carried out using the PrimeScript RT Reagent Kit with gDNA Eraser (Takara, Dalian, China) according to the manufacturer’s protocols. qRT-PCR was performed on an ABI7500 Real-Time PCR System (ABI, Vernon, CA, USA) using SYBR Green qPCR Master Mix (DBI, München, Germany). Each experiment was conducted three times, and the melting curve analysis was carried out to determine the amplification specificity. The relative expressions of tested genes were assessed using the 2−ΔΔCt method [102]. Significant differences were determined using GraphPad V5.0. The correlation between the gene expression levels of control and NaCl-treated samples was determined using the Student’s *t*-test.

## 5. Conclusions

In the present study, we conducted a transcriptome analysis on short-term acclimation to salt stress (200 mM NaCl for 24 h), and the mechanisms underlying the salt stress response in *B. distachyon* were revealed. There were 4116 DEGs in *B. distachyon* under salt stress according to the RNA-Seq analysis, including 3920 upregulated and 996 downregulated ones. Some metabolic pathways and key genes associated with cell wall and wax biosynthesis, as well as plant hormones, such as AUXIN, ABA, BRs, and active oxygen scavenging, were identified by GO terms, MapMan, and KEGG functional enrichment assays, providing some molecular tracks for understanding the response mechanism of salt stress. Our findings suggested that, in addition to the active oxygen scavenging system and osmoregulatory substances, *B. distachyon* could reduce nonstomatal water loss through the cell wall and leaf epidermal wax. Meanwhile, the key genes of the ABA signaling pathway induced stomatal closure and reduced water loss to alleviate salt-stress-induced damage.

## Figures and Tables

**Table 1:** Summary of mapping transcriptome reads to reference sequence.

Sample Name	Sample Description	Total Reads	Total Mapped	Mapped Ratio of Mapped Reads	Q30
CK_1_S	Control replication 1	59,206,904	56,875,374	96.06%	95.18%
CK_2_S	Control replication 2	63,617,088	60,841,214	95.64%	95.2%
CK_3_S	Control replication 3	57,536,132	55,180,960	95.91%	95.26%
SALT_1_S	Salt stress replication 1	55,294,072	53,503,005	96.76%	94.68%
SALT_2_S	Salt stress replication 2	65,989,512	63,143,273	95.69%	95%
SALT_3_S	Salt stress replication 3	47,315,154	45,175,264	95.48%	94.49%

**Table 2:** The top 30 dysregulated genes in *B. distachyon* under 200 mM NaCl treated and control conditions.

GENE_ID	L2fc	Padj	BP Description
Up_regulated			
BRADI2G30490	4.697967282	2.88 × 10^−52^	
BRADI3G35310	3.591061835	8.98 × 10^−38^	oxidation–reduction process
BRADI2G52155	6.171188364	1.49 × 10^−34^	
BRADI1G34670	3.484127137	9.73 × 10^−34^	
BRADI4G18910	3.643708291	1.86 × 10^−30^	
BRADI1G04690	3.443854612	4.47 × 10^−30^	
BRADI3G20677	9.241758235	2.61 × 10^−29^	oxidation–reduction process
BRADI1G59120	4.055819532	3.72 × 10^−28^	
BRADI2G21340	5.343143653	1.36 × 10^−27^	
BRADI2G37720	2.567633743	1.28 × 10^−26^	
BRADI1G44920	6.810598815	2.53 × 10^−26^	
BRADI3G49310	5.355939545	2.80 × 10^−26^	
BRADI3G19620	2.376259459	1.10 × 10^−24^	
BRADI1G10150	1.888142734	5.54 × 10^−24^	
BRADI4G01200	1.607591434	5.69 × 10^−24^	metabolic process
Down_regulated			
BRADI3G53660	−3.245924936	4.07 × 10^−33^	protein phosphorylation, metabolic process
BRADI2G44820	−4.450443575	8.69 × 10^−33^	metabolic process
BRADI3G31720	−7.041083206	4.16 × 10^−31^	
BRADI3G31727	−5.153494722	5.28 × 10^−31^	
BRADI2G19540	−1.771707314	3.15 × 10^−22^	protein metabolic process
BRADI1G74980	−6.034665237	5.24 × 10^−22^	
BRADI1G53680	−7.436218542	4.34 × 10^−20^	ion transmembrane transport
BRADI5G02750	−2.879558911	2.44 × 10^−19^	transmembrane transport
BRADI2G22520	−8.740289045	9.92 × 10^−19^	ion transmembrane transport
BRADI2G33110	−3.576800337	7.04 × 10^−18^	ion transmembrane transport
BRADI1G60250	−3.534466753	1.86 × 10^−17^	
BRADI2G44830	−2.884382952	2.90 × 10^−17^	protein phosphorylation, metabolic process
BRADI2G33950	−5.142993438	2.91 × 10^−17^	
BRADI2G19360	−1.97415848	5.62 × 10^−17^	
BRADI3G06320	−5.743267815	1.17 × 10^−15^	oxidation–reduction process

Note: The top 30 dysregulated genes with the lowest *p*-value (pval) are represented; L2fc indicates the log2FoldChange of genes differently expressed in 200 mM NaCl treated samples and control samples; padj is the adjusted *p*-value; BP Description means descriptions of genes’ potential participating biological process predicted by sequence similarity search.

**Table 3:** Top30 biological processes enriched by the upregulated and downregulated genes.

GO ID	Term	*p* Value
Up_regulated		
GO:0009834	plant-type secondary cell wall biogenesis	4.20 × 10^−9^
GO:0042744	hydrogen peroxide catabolic process	7.50 × 10^−8^
GO:0055114	oxidation-reduction process	3.20 × 10^−7^
GO:0046274	lignin catabolic process	8.60 × 10^−6^
GO:0010030	positive regulation of seed germination	3.60 × 10^−5^
GO:0006979	response to oxidative stress	4.30 × 10^−5^
GO:0007017	microtubule-based process	4.40 × 10^−5^
GO:0034605	cellular response to heat	8.20 × 10^−5^
GO:0010143	cutin biosynthetic process	1.1 × 10^−4^
GO:0030244	cellulose biosynthetic process	1.70 × 10^−4^
GO:0048640	negative regulation of developmental growth	1.90 × 10^−4^
GO:0006869	lipid transport	2.00 × 10^−4^
GO:0045492	xylan biosynthetic process	2.20 × 10^−4^
GO:0070592	cell wall polysaccharide biosynthetic process	2.30 × 10^−4^
GO:0097435	supramolecular fiber organization	2.40 × 10^−4^
Down_regulated		
GO:0034605	cellular response to heat	2.10 × 10^−7^
GO:0010030	positive regulation of seed germination	1.40 × 10^−6^
GO:0071577	zinc ion transmembrane transport	7.80 × 10^−5^
GO:0042744	hydrogen peroxide catabolic process	8.70 × 10^−5^
GO:0006414	translational elongation	2.10 × 10^−4^
GO:0009405	pathogenesis	5.60 × 10^−4^
GO:0010106	cellular response to iron ion starvation	5.60 × 10^−4^
GO:0034620	cellular response to unfolded protein	1.10 × 10^−3^
GO:0071486	cellular response to high light intensity	1.42 × 10^−3^
GO:0006826	iron ion transport	1.66 × 10^−3^
GO:0071668	plant-type cell wall assembly	1.83 × 10^−3^
GO:0000302	response to reactive oxygen species	2.44 × 10^−3^
GO:0071492	cellular response to UV-A	2.46 × 10^−3^
GO:0044264	cellular polysaccharide metabolic process	2.83 × 10^−3^
GO:0010045	response to nickel cation	4.98 × 10^−3^

**Table 4:** The TF family and their contained dysregulated gene number.

TF Family	Up Gene Num.	Down Gene Num.
bHLH	34	14
MYB	36	4
AP2/ERF	19	6
HB	15	3
bZIP	12	3
NAC	9	6
C2C2	9	4
C2H2	9	4
WRKY	7	4
OFP	10	0
Tify	8	0
GARP	2	5
HSF	1	6
B3	6	0
GRAS	2	4
C3H	3	2
MADS	3	2
TCP	5	0
Trihelix	5	0

Note: Up gene num. = Upregulated gene number; Down gene num. = Downregulated gene number.

## Supplementary Materials

The following are available online at https://www.mdpi.com/2223-7747/9/11/1522/s1, Figure S1. REVIGO analysis results for genes downregulated in *B. distachyon* under salt stress, Figure S2. ABA and BRs signal transduction pathway, Table S1: Information of the 3120 upregulated and 996 downregulated unigenes in *B. distachyon*, Table S2: GO enrichment analysis of genes differentially expressed in *B. distachyon* under salt stress, Table S3: The MapMan pathways enrichment results of the dysregulated genes in *B. distachyon*, Table S4: KEGG enrichment analysis of genes differentially expressed in *B. distachyon* under salt stress, Table S5: Transcription factors differentially expressed under salt stress in *B. distachyon*, Table S6: The information of the qRT–PCR primers.

## References

[1] Wang R., Wang X., Liu K., Zhang X., Zhang L., Fan S. (2020). Comparative transcriptome analysis of halophyte *Zoysia macrostachya* in response to salinity stress. Plants.

[2] Wang N., Qian Z., Luo M., Fan S., Zhang X., Zhang L. (2018). Identification of salt stress responding genes using transcriptome analysis in green alga *Chlamydomonas reinhardtii*. Int. J. Mol. Sci..

[3] Zhang X., Yao Y., Li X., Zhang L., Fan S. (2020). Transcriptomic analysis identifes novel genes and pathways for salt stress responses in *Suaeda salsa* leaves. Sci. Rep..

[4] Flowers T.J., Colmer T.D. (2008). Salinity tolerance in halophytes. New Phytol..

[5] Yuan F., Lyu M.-J.A., Leng B., Zhu X., Wang B. (2016). The transcriptome of NaCl-treated *Limonium bicolor* leaves reveals the genes controlling salt secretion of salt gland. Plant Mol. Biol..

[6] Ahmed M.Z., Shimazaki T., Gulzar S., Kikuchi A., Gul B., Khan M.A., Koyro H.W., Huchzermeyer B., Watanabe K.N. (2013). The influence of genes regulating transmembrane transport of Na^+^ on the salt resistance of *Aeluropus lagopoides*. Funct. Plant Biol..

[7] Zelm E.V., Zhang Y., Testerink C. (2020). Salt tolerance mechanisms of plants. Ann. Rev. Plant Biol..

[8] Gong Z., Xiong L., Shi H., Yang S., Herrera-Estrella L.R., Xu G., Chao D., Li J., Wang P., Qin F. (2020). Plant abiotic stress response and nutrient use efficiency. Sci. China Life Sci..

[9] Sudhir P., Murthy S.D.S. (2004). Effects of salt stress on basic processes of photosynthesis. Photosynthetica.

[10] Donaldsona L., Ludidib N., Knightc M.R., Gehringb C., Denbya K. (2004). Salt and osmotic stress cause rapid increases in *Arabidopsis thaliana* cGMP levels. FEBS Lett..

[11] Kiegle E., Moore C.A., Haseloff J., Tester M.A., Knight M.R. (2000). Cell-type-specific responses to drought, salt and cold in the Arabidopsis root. Plant J..

[12] Gobert A., Isayenkov S., Voelker C., Czempinski K., Maathuis F.J.M. (2007). The two-pore channel TPK1 gene encodes the vacuolar K^+^ conductance and plays a role in K^+^ homeostasis. Proc. Natl. Acad. Sci. USA.

[13] Latz A., Mehlmer N., Zapf S., Mueller T.D., Wurzinger B., Pfister B., Csaszar E., Hedrich R., Teige M., Becker D. (2013). Salt stress triggers phosphorylation of the *Arabidopsis* vacuolar K^+^ channel TPK1 by Calcium-Dependent Protein Kinases (CDPKs). Mol. Plant.

[14] Hadi M.R., Karimi N. (2012). The role of calcium in plant’s salt tolerance. J. Plant Nutr..

[15] Wang F., Zhu H., Chen D., Li Z., Peng R., Yao Q. (2016). A grape bHLH transcription factor gene, VvbHLH1, increases the accumulation of flavonoids and enhances salt and drought tolerance in transgenic *Arabidopsis thaliana*. Plant Cell Tissue Organ Cult..

[16] Yang A., Dai X., Zhang W. (2012). A R2R3-type MYB gene, OsMYB2, is involved in salt, cold, and dehydration tolerance in rice. J. Exp. Bot..

[17] Hong Y., Zhang H., Huang L., Li D., Song F. (2016). Overexpression of a stress-responsive NAC transcription factor gene ONAC022 improves drought and salt tolerance in rice. Front. Plant Sci..

[18] Zhang W., Liu S., Li C., Zhang P., Zhang P. (2019). Transcriptome sequencing of Antarctic moss under salt stress emphasizes the important roles of the ROS-scavenging system. Gene.

[19] Li Z., Li J., Bing J., Zhang G. (2019). The role analysis of APX gene family in the growth and developmental processes and in response to abiotic stresses in *Arabidopsis thaliana*. Hereditas (Beijing).

[20] Sui N., Tian S., Wang W., Wang M., Fan H. (2017). Overexpression of glycerol-3-phosphate acyltransferase from *Suaeda salsa* improves salt tolerance in Arabidopsis. Front. Plant Sci..

[21] Kito K., Yamane K., Yamamori T., Matsuhira H., Tanaka Y., Takabe T. (2018). Isolation, functional characterization and stress responses of raffinose synthase genes in sugar beet. J. Plant Biochem. Biotechnol..

[22] Barnawal D., Bharti N., Pandey S.S., Pandey A., Kalra A. (2017). Plant growth-promoting rhizobacteria enhance wheat salt and drought stress tolerance by altering endogenous phytohormone levels and TaCTR1/TaDREB2 expression. Physiol. Plant..

[23] Wu Q., Wang M., Shen J., Chen D., Zheng Y., Zhang W. (2018). ZmOST1 mediates ABA regulation of guard cell ion channels and drought stress responses. J. Integr. Plant Biol..

[24] Askari-Khorasgani O., Pessarakli M. (2019). Chapter 19 Phytohormone homeostasis and crosstalk effects in response to osmotic stress. Handbook of Plant and Crop Stress.

[25] WasKiewicz A., Beszterda M., Goliński P., Ahmad P., Azooz M.M., Prasad M.N.V. (2013). Salt Stress in Plants.

[26] Pandey V., Bhatt I.D., Nandi S.K., Khan M.I.R., Reddy P.S., Ferrante A., Khan N.A. (2019). Role and regulation of auxin signaling in abiotic stress tolerance. Plant Signaling Molecules.

[27] Li S., An Y., Hailati S., Zhang J., Yang P. (2019). Overexpression of the cytokinin oxidase/dehydrogenase (CKX) from *Medicago sativa* enhanced salt stress tolerance of Arabidopsis. J. Plant Biol..

[28] Krishna P., Prasad B.D., Rahman T. (2017). Brassinosteroid action in plant abiotic stress tolerance. Methods in Molecular Biology.

[29] Ma J., Gao X., Liu Q., Yun S., Zhang D., Jiang L., Li C. (2017). Overexpression of TaWRKY146 increases drought tolerance through inducing stomatal closure in *Arabidopsis thaliana*. Front. Plant Sci..

[30] Wang W., Liu X., Gai X., Ren J., Liu X., Cai Y., Wang Q., Ren H. (2015). *Cucumis sativus* L. WAX2 plays a pivotal role in wax biosynthesis, influencing pollen fertility and plant biotic and abiotic stress responses. Plant Cell Physiol..

[31] Mockler T.C., Schmutz J., Rokhsar D., Bevan M.W., Barry K., Lucas S., Harmon-Smith M., Lail K., Vogel J.P., Garvin D.F. (2010). Genome sequencing and analysis of the model grass *Brachypodium distachyon*. Nature.

[32] Kellogg E.A. (2015). Flowering Plants. Monocots.

[33] Vogel J.P., Garvin D.F., Leong O.M., Hayden D.M. (2006). Agrobacterium-mediated transformation and inbred line development in the model grass *Brachypodium distachyon*. Plant Cell Tissue Organ Cult..

[34] Draper J., Mur L.A.J., Jenkins G., Ghosh-Biswas G.C., Bablak P., Hasterok R., Routledge A.P.M. (2001). *Brachypodium distachyon*. A New Model System for Functional Genomics in Grasses. Plant Physiol..

[35] Wang C., Wang Y., Pan Q., Chen S., Feng C., Hai J., Li H. (2019). Comparison of Trihelix transcription factors between wheat and *Brachypodium distachyon* at genome-wide. BMC Genom..

[36] Kang H., Zhang M., Zhou S., Guo Q., Chen F., Wu J., Wang W. (2016). Overexpression of wheat ubiquitin gene, Ta-Ub2, improves abiotic stress tolerance of *Brachypodium distachyon*. Plant Sci..

[37] Lv D., Subburaj S., Cao M., Yan X., Li X., Appels R., Sun D., Ma W., Yan Y. (2014). Proteome and phosphoproteome characterization reveals new response and defense mechanisms of *Brachypodium distachyon* leaves under salt stress. Mol. Cell. Proteom..

[38] You J., Zhang L., Song B., Qi X., Chan Z. (2015). Systematic analysis and identification of stress-responsive genes of the NAC gene family in *Brachypodium distachyon*. PLoS ONE.

[39] Wang L., Hu W., Sun J., Liang X., Yang X., Wei S., Wang X., Zhou Y., Xiao Q., Yang G. (2015). Genome-wide analysis of SnRK gene family in *Brachypodium distachyon* and functional characterization of BdSnRK2.9. Plant Sci..

[40] Zhou Y., Yang P., Cui F., Zhang F., Luo X., Xie J. (2016). Transcriptome analysis of salt stress responsiveness in the seedlings of Dongxiang wild rice (*Oryza rufipogon* Griff.). PLoS ONE.

[41] Luo Q., Teng W., Fang S., Li H., Li B., Chu J., Li Z., Zheng Q. (2019). Transcriptome analysis of salt-stress response in three seedling tissues of common wheat. Crop J..

[42] Yang Z., Zheng H., Wei X., Song J., Wang B., Sui N. (2018). Transcriptome analysis of sweet Sorghum inbred lines differing in salt tolerance provides novel insights into salt exclusion by roots. Plant Soil.

[43] Owen H.J. (1988). Role of abscisic acid in a Ca^2+^ second messenger system. Physiol. Plant..

[44] Ma L., Ye J., Yang Y., Lin H., Yue L., Luo J., Long Y., Fu H., Liu X., Zhang Y. (2019). The SOS2-SCaBP8 complex generates and fine-tunes an AtANN4-dependent calcium signature under salt stress. Dev. Cell.

[45] Zhu J. (2016). Abiotic stress signaling and responses in plants. Cell.

[46] Ren X., Qi G., Feng H., Zhao S., Wu W. (2013). Calcineurin B-like protein CBL10 directly interacts with AKT1 and modulates K^+^ homeostasis in Arabidopsis. Plant J..

[47] Kim D.Y., Hong M.J., Jang J.H., Seo Y.W. (2012). cDNA-AFLP analysis reveals differential gene expression in response to salt stress in *Brachypodium distachyon*. Genes Genom..

[48] Sade N., Maria D.M.R.W., Ke X., Brotman Y., Wright M., Khan I., De Souza W., Bassil E., Tobias C.M., Thilmony R. (2018). Salt tolerance of two perennial grass *Brachypodium sylvaticum* accessions. Plant Mol. Biol..

[49] Zhang J., Feng J., Lu J., Yang Y., Zhang X., Wan D., Liu J. (2014). Transcriptome differences between two sister desert poplar species under salt stress. BMC Genom..

[50] Liu Q., Liu R., Ma Y., Song J. (2018). Physiological and molecular evidence for Na^+^ and Cl^−^ exclusion in the roots of two *Suaeda salsa* populations. Aquat. Bot..

[51] Yao Y., Zhang X., Wang N., Cui Y., Zhang L., Fan S. (2020). Transcriptome analysis of salt stress response in halophyte *Atriplex centralasiatica* leaves. Acta Physiol. Plant..

[52] Miller G., Suzuki N., Ciftci-Yilmaz S., Mittler R. (2010). Reactive oxygen species homeostasis and signalling during drought and salinity stresses. Plant Cell Environ..

[53] Qi W., Xue B., Wei Z., Dabing X., Yan W., Jun Y., Liang Z., Gang Z. (2017). De novo assembly and analysis of tartary buckwheat (*Fagopyrum tataricum* Garetn.) transcriptome discloses key regulators involved in salt-stress response. Genes.

[54] Chan C., Lam H.-M. (2014). A putative lambda class glutathione S-transferase enhances plant survival under salinity stress. Plant Cell Physiol..

[55] Jha B., Sharma A., Mishra A. (2011). Expression of SbGSTU (tau class glutathione S-transferase) gene isolated from *Salicornia brachiata* in tobacco for salt tolerance. Mol. Biol. Rep..

[56] Long L., Yang W.W., Liao P., Guo Y., Kumar A., Gao W. (2019). Transcriptome analysis reveals differentially expressed ERF transcription factors associated with salt response in cotton. Plant Sci..

[57] Zhu N., Cheng S., Liu X., Du H., Dai M. (2015). The R2R3-type MYB gene OsMYB91 has a function in coordinating plant growth and salt stress tolerance in rice. Plant Sci..

[58] Zhang L., Zhao G., Xia C., Jia J., Liu X., Kong X. (2012). A wheat R2R3-MYB gene, TaMYB30-B, improves drought stress tolerance in transgenic Arabidopsis. J. Exp. Bot..

[59] Sakuma Y., Liu Q., Dubouzet J.G., Abe H., Shinozaki K., Yamaguchi-Shinozaki K. (2002). DNA-binding specificity of the ERF/AP2 domain of arabidopsis DREBs, transcription factors involved in dehydration- and cold-inducible gene expression. Biochem. Biophys. Res. Commun..

[60] Zhao K. (1993). Salt Tolerance Physiology of Plants.

[61] Storey R., Jones R.W. (1978). Salt stress and comparative physiology in the Gramineae. III. Effect of salinity upon ion relations and glycinebetaine and proline levels in *Spartina* × *townsendii*. Aust. J. Plant Physiol..

[62] Amaral M.N.D., Arge L.W.P., Benitez L.C., Danielowski R., Silveira S.F.d.S., Farias D.D.R., Oliveira A.C.D., Maia L.C.D., Braga E.J.B. (2016). Comparative transcriptomics of rice plants under cold, iron, and salt stresses. Funct. Integr. Genom..

[63] Sun Z., Qi X., Wang Z., Li P., Zhao Y. (2013). Overexpression of TsGOLS2, a galactinol synthase, in *Arabidopsis thaliana* enhances tolerance to high salinity and osmotic stresses. Plant Physiol. Biochem..

[64] Lahuta L.B., Górecki R.J. (2011). Raffinose in seedlings of winter vetch (*Vicia villosa* Roth.) under osmotic stress and followed by recovery. Acta Physiol. Plant..

[65] Pluskota W.E., Szablińska J., Obendorf R.L., Górecki R.J., Lahuta L.B. (2015). Osmotic stress induces genes, enzymes and accumulation of galactinol, raffinose and stachyose in seedlings of pea (*Pisum sativum* L.). Acta Physiol. Plant..

[66] Vardhini B.V., Anjum N.A. (2015). Brassinosteroids make plant life easier under abiotic stresses mainly by modulating major components of antioxidant defense system. Front. Environ. Sci..

[67] Christian Z., Geilfus C.M., Mühling K.H., Jutta L.-M. (2013). The influence of salt stress on ABA and auxin concentrations in two maize cultivars differing in salt resistance. J. Plant Physiol..

[68] Liu W., Li R., Han T., Cai W., Fu Z., Lu Y. (2015). Salt stress reduces root meristem size by nitric oxide-mediated modulation of auxin accumulation and signaling in *Arabidopsis*. Plant Physiol..

[69] Qiu T., Qi M., Ding X., Zheng Y., Zhou T., Chen Y., Han N., Zhu M., Bian H., Wang J. (2019). The SAUR41 subfamily of SMALL AUXIN UP RNA genes is abscisic acid inducible to modulate cell expansion and salt tolerance in *Arabidopsis thaliana* seedlings. Ann. Bot..

[70] Sarmad J., Shariati M., Haghjou M.M. (2007). Relationship between endogenous abscisic acid and B-carotene synthesis in unicellular green Alga *Dunaliella*. Am. Eurasian J. Agric. Environ. Sci..

[71] Zhang M., Zhao J. (2017). The Arabidopsis U-box E3 ubiquitin ligase PUB30 negatively regulates salt tolerance by facilitating BRI1 kinase inhibitor 1 (BKI1) degradation. Plant Cell Environ..

[72] Sahni S., Prasad B.D., Liu Q., Grbic V., Sharpe A., Singh S.P., Krishna P. (2016). Overexpression of the brassinosteroid biosynthetic gene DWF4 in *Brassica napus* simultaneously increases seed yield and stress tolerance. Sci. Rep..

[73] Li Z.Y., Xu Z.S., He G.Y., Yang G.X., Chen M., Li L.C., Ma Y.Z. (2012). A mutation in Arabidopsis BSK5 encoding a brassinosteroid-signaling kinase protein affects responses to salinity and abscisic acid. Biochem. Biophys. Res. Commun..

[74] Kerstiens G., Tych W., Robinson M.F., Mansfield T.A. (2002). Special Issue: Stomata. Sodium-Related Partial Stomatal Closure and Salt Tolerance of *Aster tripolium*. New Phytol..

[75] Kerstiens G. (1996). Cuticular water permeability and its physiological significance. J. Exp. Bot..

[76] Yan H., Shah S.S., Zhao W., Liu F. (2020). Variations in water relations, stomatal characteristics, and plant growth between quinoa and pea under salt-stress conditions. Pak. J. Bot..

[77] Zhao Y., Zhao S., Mao T., Qu X., Cao W., Zhang L., Zhang W., He L., Li S., Ren S. (2011). The Plant-specific actin binding protein SCAB1 stabilizes actin filaments and regulates stomatal movement in Arabidopsis. Plant Cell.

[78] Sierla M., Hõrak H., Overmyer K., Waszczak C., Yarmolinsky D., Maierhofer T., Vainonen J.P., Salojärvi J., Denessiouk K., Laanemets K. (2018). The receptor-like pseudokinase GHR1 is required for stomatal closure. Plant Cell.

[79] Cunxu W., Jianbo W., Yifang C., Weidong Z., Guorong S. (2004). Epicuticular wax of leaf epidermis: A functional structure for salt excretion in a halophyte *Puccinellia tenuiflora*. Acta Ecol. Sin..

[80] Fahn A. (1988). Secretory tissues in vascular plants. New Phytol..

[81] Wang J., Meng Y., Li B., Ma X., Lai Y., Si E., Yang K., Xu X., Shang X., Wang H. (2015). Physiological and proteomic analyses of salt stress response in the halophyte *Halogeton glomeratus*. Plant Cell Environ..

[82] Morel F., Lucarain C. (1967). A flame spectrophotometer to determine sodium and potassium in biological samples of the nanoliter order. J. Physiol..

[83] Gama P.B.S., Tanaka K., Eneji A.E., Eltayeb A.E., Siddig K.E. (2009). Salt-induced stress effects on biomass, photosynthetic rate, and reactive oxygen species-scavenging enzyme accumulation in common bean. J. Plant Nutr..

[84] Chance B., Maehly A.C. (1955). The assay of catalases and peroxidases. Methods Enzymol..

[85] Tomita K. (1983). Assay of Catalase Activity Based on the Absorption of Ultraviolet Light by H_2_O_2_.

[86] Dubois M., Gilles K.A., Hamilton J.K., Rebers P.A., Smith F. (1956). Colorimetric method for determination of sugars and related substances. Anal. Chem..

[87] Xie B., Rao Z., Xiang J., Yang Y., Yuan J., Yin J., Yang Y., Yi M., Cao Y. (2015). Evaluation of uncertainty of the determination of soluble sugar in tobacco by anthrone colorimetry. China Meas. Test.

[88] Ábrahám E., Hourton-Cabassa C., Erdei L., Szabados L. (2010). Methods for determination of proline in plants. Plant Stress Toler..

[89] Kim D., Langmead B., Salzberg S.L. (2015). HISAT: A fast spliced aligner with low memory requirements. Nat. Methods.

[90] Pertea M., Pertea G.M., Antonescu C.M., Chang T.-C., Mendell J.T., Salzberg S.L. (2015). StringTie enables improved reconstruction of a transcriptome from RNA-seq reads. Nat. Biotechnol..

[91] Liao Y., Smyth G.K., Shi W. (2013). FeatureCounts: An efficient general purpose program for assigning sequence reads to genomic features. Bioinformatics.

[92] Trapnell C., Williams B.A., Pertea G., Mortazavi A., Kwan G., Van Baren M.J., Salzberg S.L., Wold B.J., Pachter L. (2010). Transcript assembly and quantification by RNA-Seq reveals unannotated transcripts and isoform switching during cell differentiation. Nat. Biotechnol..

[93] Love M.I., Huber W., Anders S. (2014). Moderated estimation of fold change and dispersion for RNA-seq data with DESeq2. Genome Biol..

[94] Robinson M.D., Mccarthy D.J., Smyth G.K. (2010). edgeR: A Bioconductor package for differential expression analysis of digital gene expression data. Bioinformatics.

[95] Bel M.V., Diels T., Vancaester E., Kreft L., Botzki A., Peer Y.V.D., Coppens F., Vandepoele K. (2018). PLAZA 4.0: An integrative resource for functional, evolutionary and comparative plant genomics. Nucleic Acids Res..

[96] Alexa A., Rahnenfuhrer J. (2006). topGO: Enrichment analysis for Gene Ontology; R Package Version. https://rdrr.io/bioc/topGO/.

[97] Supek F., Bošnjak M., Škunca N., Šmuc T. (2011). REVIGO summarizes and visualizes long lists of Gene Ontology terms. PLoS ONE.

[98] Wu J., Mao X., Tao C., Luo J., Wei L. (2006). KOBAS server: A web-based platform for automated annotation and pathway identification. Nucleic Acids Res..

[99] Thimm O., Bläsing O., Gibon Y., Nagel A., Meyer S., Krüger P., Selbig J., Müller L.A., Rhee S.Y., Stitt M. (2004). Mapman: A user-driven tool to display genomics data sets onto diagrams of metabolic pathways and other biological processes. Plant J..

[100] Zheng Y., Jiao C., Sun H., Rosli H.G., Pombo M.A., Zhang P., Banf M., Dai X., Martin G.B., Giovannoni J.J. (2016). iTAK: A program for genome-wide prediction and classification of plant transcription factors, transcriptional regulators, and protein kinases. Mol. Plant.

[101] Hong S.Y., Seo P.J., Yang M.-S., Xiang F., Park C.M. (2008). Exploring valid reference genes for gene expression studies in *Brachypodium distachyon* by real-time PCR. BMC Plant Biol..

[102] Yuan J.S., Reed A., Chen F., Stewart C.N. (2006). Statistical analysis of real-time PCR data. BMC Bioinform..

