# Supplementary figures and images for: Screening of Salt Stress Responsive Genes in Brachypodium distachyon (L.) Beauv. by Transcriptome Analysis
Source: Plants (Basel). 2020 Nov 9;9(11):1522. doi: 10.3390/plants9111522 (PMC7697870; doi:10.3390/plants9111522)

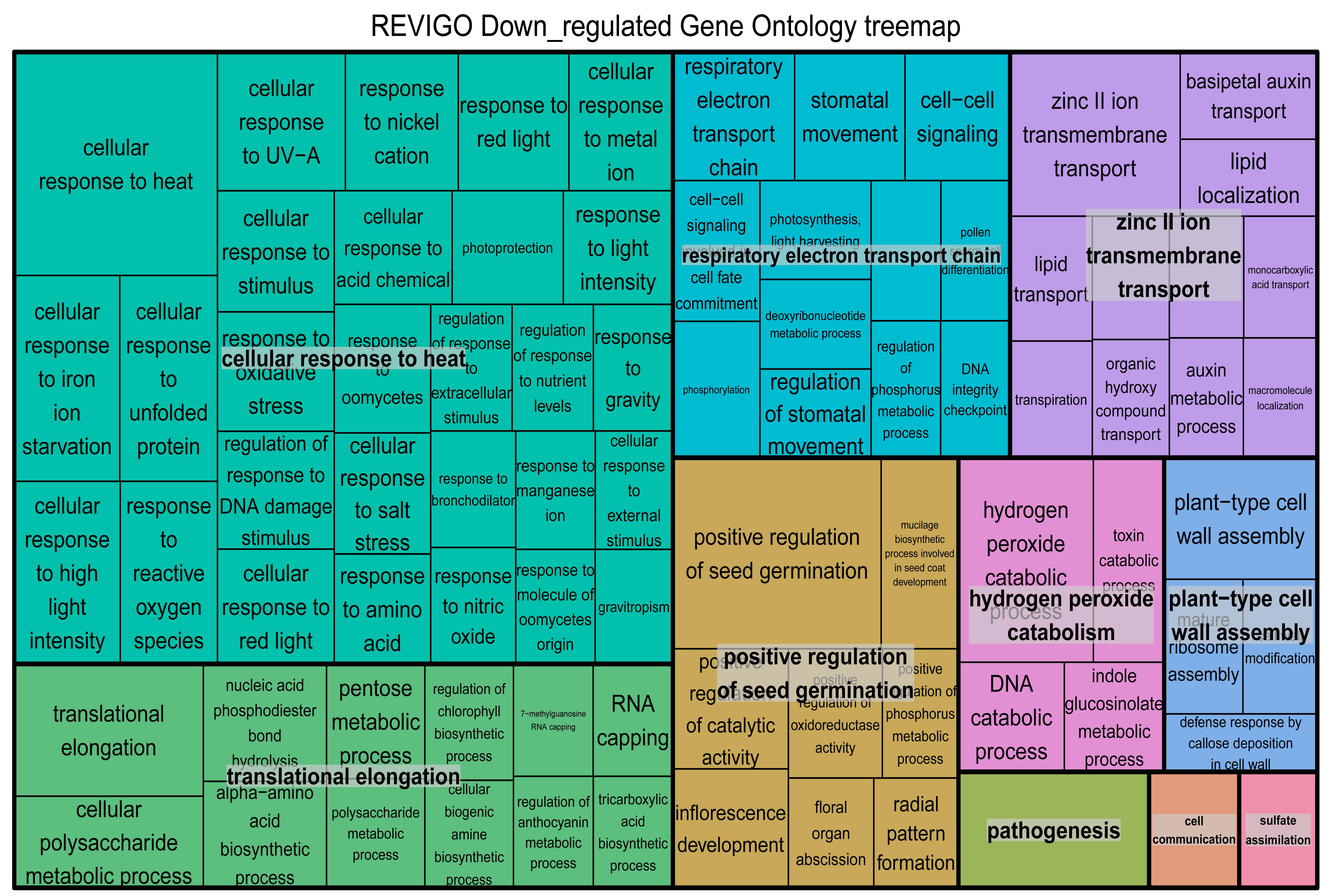

Supplement: Supplementary file 1 [file plants-09-01522-s001.zip › Supplementary materials/Figure S1. REVIGO analysis results for genes downregulated in B. distachyon under salt stress.jpg]

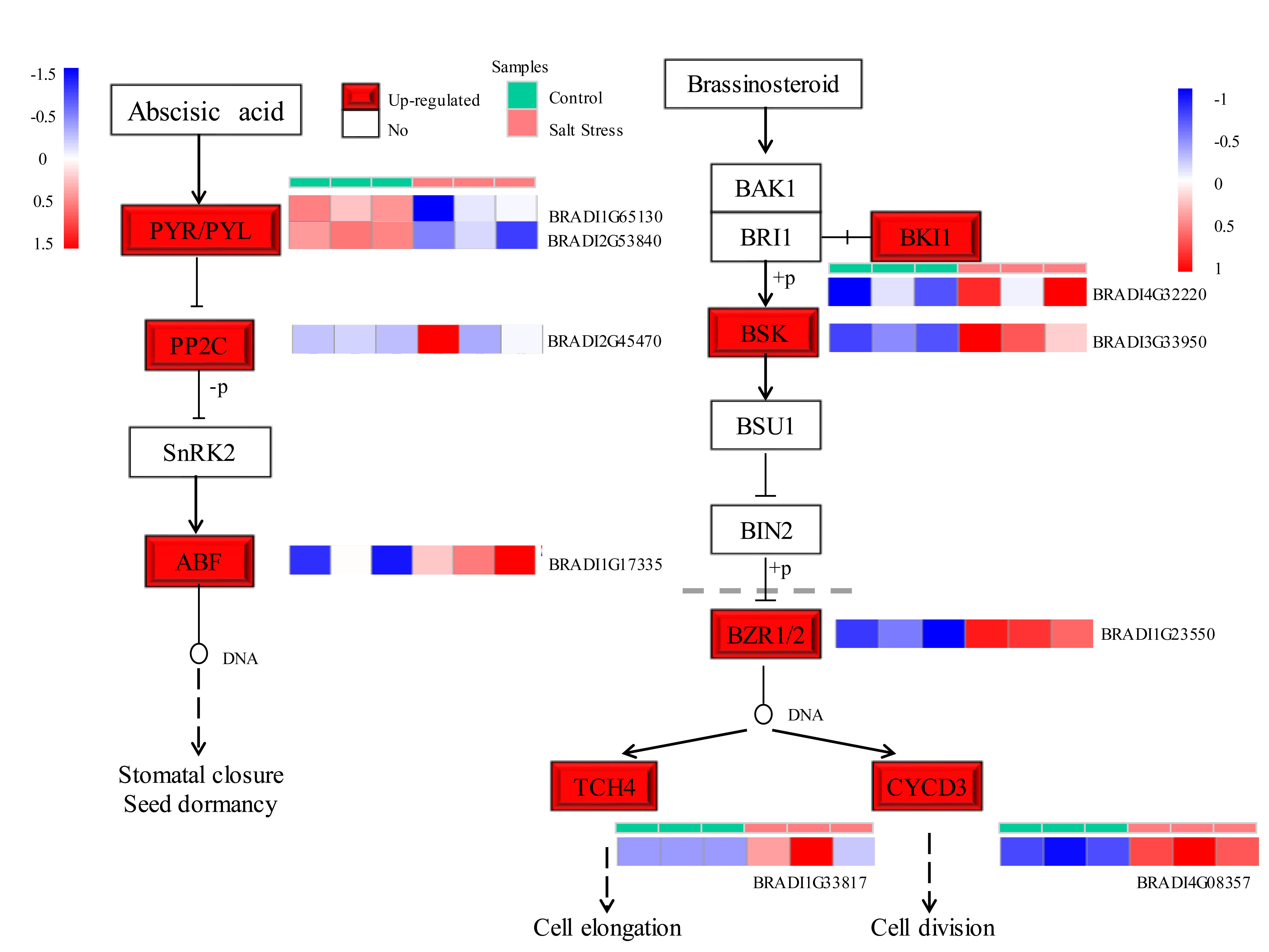

Supplement: Supplementary file 1 [file plants-09-01522-s001.zip › Supplementary materials/Figure S2. ABA and BRs signal transduction pathway.jpg]
